# Association between intrinsic capacity trajectories and risk of stroke incidence in middle-aged and older chinese adults: Evidence from a nationwide prospective cohort study based on CHARLS

**DOI:** 10.1371/journal.pone.0342480

**Published:** 2026-03-04

**Authors:** Sumei Zhou, Min Chen, Shirong Shao, Zhi Zeng

**Affiliations:** 1 Department of Neurosurgery, Deyang People’s Hospital, Deyang, Sichuan, China; 2 Department of Gastroenterology, Deyang People’s Hospital, Deyang, Sichuan, China; Universite de Liege, BELGIUM

## Abstract

**Background:**

Stroke is a major public health concern and a leading cause of disability and death in aging populations. Intrinsic capacity (IC), a concept introduced by the World Health Organization, reflects an individual's overall functional ability across multiple domains including cognition, psychological well-being, mobility, vitality, and sensory function. IC has emerged as a core metric within the healthy aging framework, but its prospective relationship with stroke risk remains unclear. A deeper understanding of this link may inform early, function-based prevention strategies.

**Methods:**

This study used data from 10,751 participants aged 45 years or older from the China Health and Retirement Longitudinal Study (CHARLS). Cox proportional hazards models were used to estimate the association between IC and incident stroke, with stepwise adjustment for demographic, behavioral, and health-related covariates. Modeling IC as a continuous variable enabled examination of linear trends, while quartile-based classification allowed evaluation of potential non-linear associations and improved interpretability. Kaplan–Meier curves and log-rank tests were used to compare stroke-free survival across IC quartiles. Restricted cubic spline analysis was performed to explore the presence of a non-linear association between IC and stroke risk. Robustness was tested through sensitivity analyses excluding participants with baseline cognitive impairment and those aged ≥80 years. Statistical analyses were conducted using Stata and R.

**Results:**

Over a 7-year follow-up, 243 participants (2.26%) experienced incident stroke. Stroke incidence decreased progressively with increasing IC levels, from 4.84% in the lowest quartile to 0.46% in the highest. Kaplan–Meier analysis showed significantly lower cumulative stroke incidence among individuals with higher IC (log-rank p < 0.001). In fully adjusted Cox models, each one-point increase in IC was associated with a 35.1% reduction in stroke risk (HR = 0.649; 95% CI: 0.599–0.702). Compared to the lowest IC quartile, the highest quartile had an 89.6% lower stroke risk (HR = 0.104; 95% CI: 0.055–0.197). Restricted cubic spline models confirmed a predominantly linear inverse association, with a steeper risk gradient at lower IC levels. Subgroup analyses revealed stronger protective associations in women, older adults (≥60 years), urban residents, and non-smokers or non-drinkers. Results remained consistent across all sensitivity analyses.

**Conclusions:**

Higher IC was independently associated with a significantly reduced risk of incident stroke, underscoring IC's potential as a holistic, function-based indicator of cerebrovascular vulnerability. These findings provide empirical support for the World Health Organization's healthy aging framework, emphasizing IC as a modifiable reserve that reflects early, multidomain functional decline before clinical disease onset. Incorporating IC into routine screening and prevention strategies may enhance early identification of high-risk individuals and enable more targeted, function-oriented interventions, thereby promoting healthy aging and helping to reduce the future burden of stroke.

## 1. Introduction

Stroke is the second leading cause of death and a major contributor to long-term disability worldwide, posing substantial challenges to public health systems and socioeconomic development [1]. According to the Global Burden of Disease (GBD) 2021 report, stroke accounted for 10.7% of global deaths and 5.6% of all disability-adjusted life years (DALYs), ranking second in mortality and fourth in total DALYs globally [2]. In China, stroke remains the leading cause of death and disability, with a growing proportion of cases occurring at younger ages among adults aged 40–64 years [3–8].

This rising stroke burden is occurring alongside rapid population aging. By 2020, more than 42% of the Chinese population was aged ≥45 years, a proportion expected to exceed 47% by 2040 [9–12]. These converging trends underscore the urgent need for early, modifiable indicators of functional decline to support stroke prevention in aging adults.

To guide healthy aging strategies, the World Health Organization (WHO) introduced the concept of Intrinsic Capacity (IC) as a central pillar of its functional-based approach. IC is a composite measure of an individual's physical and mental capacities across five domains: locomotion, vitality, sensory function, cognition, and psychological well-being [13–15]. In China, declines in one or more IC domains are common among older adults, affecting up to 74% of this population [16,17]. Loss of IC has been associated with frailty, disability, cognitive decline, reduced quality of life, hospitalization, and increased mortality risk [18–22]. Moreover, accumulating evidence suggests that lower IC may reflect early subclinical deterioration that precedes major clinical events such as cardiovascular disease and death [23–25].

From a conceptual standpoint, declining IC may serve as an integrative marker of multidomain vulnerability that is not adequately captured by traditional biomedical risk factors. Mechanistically, low IC often coexists with cognitive impairment, depressive symptoms, malnutrition, physical inactivity, and systemic inflammation—factors that are independently linked to cerebrovascular pathology [26–29]. Cognitive dysfunction may delay recognition of early stroke symptoms and hinder timely care-seeking [30]; depressive symptoms and psychosocial stress can disrupt the hypothalamic–pituitary–adrenal axis and activate proinflammatory pathways, compromising vascular integrity [31,32]. Reduced mobility and poor nutritional status may further accelerate endothelial dysfunction and atherosclerosis [33,34]. In addition, individuals with lower IC tend to have reduced engagement in health-promoting behaviors, such as physical activity, healthy eating, and chronic disease management, further amplifying stroke susceptibility. These mechanisms highlight the relevance of IC not only as a marker of functional status, but also as a potential early indicator of cerebrovascular risk.

Despite growing recognition of IC as a central construct in healthy aging, its association with stroke risk remains insufficiently explored, particularly in large, population-based longitudinal studies. Existing research has primarily focused on cross-sectional designs, single IC domains, or broader cardiovascular disease outcomes, and few studies have evaluated stroke as a distinct endpoint [35]. Moreover, evidence from nationally representative cohorts in middle-aged populations is especially limited.

To address this gap, we used data from the China Health and Retirement Longitudinal Study (CHARLS), a nationally representative cohort of adults aged ≥45 years, to examine the association between baseline IC and incident stroke. Grounded in the healthy aging framework, this study aimed to evaluate whether IC can serve as a multidimensional, function-based indicator for early stroke risk stratification, and inform function-centered prevention strategies in aging populations.

## 2. Materials and methods

### 2.1. Study design

This research utilizes data from the China Health and Retirement Longitudinal Study (CHARLS; data available at http://charls.pku.edu.cn/en), a nationwide prospective cohort initiated in 2011. Employing multistage, stratified probability sampling, CHARLS recruited 17,708 participants aged ≥45 from 150 counties/districts and 450 communities across 28 provinces. Data collection occurs via structured questionnaires every 2–3 years, with five waves completed in 2011, 2013, 2015, 2018, and 2020 [36]. The current analysis focuses on individuals aged ≥45 at baseline, with stroke outcomes assessed through 2018. CHARLS received ethical approval from the Peking University Biomedical Ethics Committee (IRB00001052‑11015), and all participants provided informed consent.

### 2.2. Participants and sample selection

**Fig 1:**
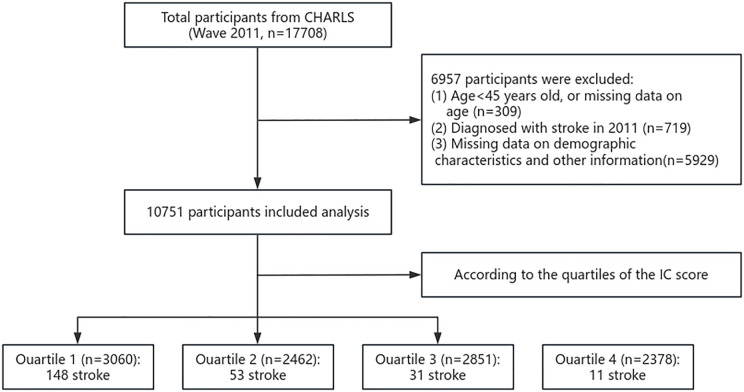
Flow chart of the study participants. Participants were categorized into quartiles based on baseline IC scores. The number of stroke cases listed in each quartile (e.g., “148 stroke” in Quartile 1) represents the number of participants within that IC quartile who experienced incident stroke during the follow-up period (2011–2020).

Participants were eligible for inclusion if they were aged 45 years or older at baseline and had complete data on IC and stroke status at a minimum of two time points. Inclusion criteria were as follows: (1) no history of stroke at baseline, (2) availability of all key analytical variables, and (3) at least one valid follow-up record. Participants with missing data on any of the five IC domains or critical covariates (e.g., age, sex, education, comorbidities) were excluded from the analysis to ensure consistent model specification and accurate IC score calculation. Continuous variables were winsorized to minimize the influence of outliers. After applying these criteria, a total of 10,751 participants with complete data were retained for the final analytic sample. Participants were categorized into four groups based on the quartiles of baseline IC scores and were followed up until 2020 (Fig 1).

### 2.3. Variable definitions

#### 2.3.1. Intrinsic capacity assessment.

IC was evaluated based on the WHO-recommended ICOPE screening tool [37], encompassing six domains: locomotion, vitality, hearing, vision, cognition, and psychological well‑being. Each intact domain scored 1 point; impairment scored 0, yielding a total IC score ranging from 0 to 6 (lower scores indicate poorer IC). Domain-specific assessments included: (1) Locomotion: Five-times sit-to-stand test; completion within 14 s indicated intact mobility. (2) Vitality: Represented by body mass index (BMI); BMI > 18.5 kg/m² was considered intact, while BMI ≤ 18.5 kg/m² indicated decreased vitality. (3) Hearing: Based on self-reported hearing quality; only “poor” responses were classified as impaired. (4) Vision: Participants rated near and far vision; any rating of “poor” qualified as visual impairment. (5) Cognition: Evaluated via immediate and delayed recall (20-word test) and orientation/calculation/figure-copy tasks (total of 11 points). Only if both assessments were intact was cognitive function considered unimpaired. (6) Psychological: Measured using the CES-D scale; scores ≥12 indicated depressive symptoms or psychological impairment.

#### 2.3.2. Stroke ascertainment.

Incident stroke, including ischemic and hemorrhagic types, was defined by self-reported new physician diagnoses during follow-up. Participants were asked: “Have you ever been told by a doctor that you had a stroke?”, “Since the last interview, have you been newly diagnosed with stroke?”, and “When were you first diagnosed?” [36,38,39]. Time-to-event was calculated from baseline to first stroke report or final follow-up.

#### 2.3.3. Covariates.

We adjusted for known stroke risk factors [40], including: Sociodemographic variables: Age, sex, education, marital status, cohabitation with children, medical and pension insurance, smoking and alcohol use, physical activity levels (light/moderate/vigorous), and self-rated health. Household factors: Household income, expenditure, household size, home ownership, and number of children.

### 2.4. Statistical analysis

All statistical analyses were conducted using Stata MP version 16.0 and R version 4.2.1. Continuous variables were tested for normality using the Kolmogorov–Smirnov test and for homogeneity of variance using Levene’s test. Descriptive statistics were reported as mean ± standard deviation (SD) for normally distributed variables, or median and interquartile range (IQR) for skewed data. Categorical variables were summarized as frequencies and percentages. Group comparisons were performed using one-way ANOVA or Kruskal–Wallis tests for continuous variables, and Chi-square tests for categorical variables.

IC was analyzed both as a continuous variable (range: 0–6) and as a categorical variable, classified into quartiles based on baseline distribution (Q1–Q4). The quartile-based classification was chosen to reflect the empirical distribution of IC and to avoid arbitrary cut-off points. Analyzing IC as a continuous variable allowed for the assessment of linear trends in stroke risk, while categorization into quartiles enabled the evaluation of potential non-linear associations.

Cox proportional hazards regression models were used to estimate the association between IC and incident stroke, with hazard ratios (HRs) and 95% confidence intervals (CIs) reported. The lowest IC quartile (Q1) was used as the reference group. A stepwise modeling strategy was adopted: Model 1 was unadjusted; Model 2 adjusted for sociodemographic variables (age, sex, education level, marital status, and residential area); and Model 3 additionally adjusted for lifestyle and clinical factors (smoking status, alcohol consumption, body mass index, chronic disease history, and depressive symptoms).

Kaplan–Meier survival curves were constructed to illustrate cumulative stroke incidence across IC quartiles, and statistical differences were assessed using the log-rank test. To further explore potential non-linear relationships between IC and stroke risk, restricted cubic spline (RCS) models were applied with IC treated as a continuous predictor. Sensitivity analyses were performed to evaluate the robustness of our findings by: (1) excluding participants with baseline cognitive impairment, and (2) excluding participants aged ≥80 years.

## 3. Results

### 3.1. Baseline characteristics

The study population characteristics at baseline were presented as in overall and quartiles of IC (Table 1). The mean age of the overall study population was 58.06 ± 8.94 years, and 5,676 participants (52.79%) were female. Participants in higher IC quartiles were generally younger, more likely to be male and married, and had higher educational attainment than those in the lowest quartile. Mean age decreased steadily from 60.48 ± 9.91 years in Quartile 1 to 55.25 ± 7.46 years in Quartile 4, while the proportion of participants with at least high school education increased from 7.74% to 22.12% across IC quartiles (p < 0.001). Higher IC was also associated with fewer children but greater household income and consumption, whereas household size did not differ significantly. Regarding health-related behaviors, the prevalence of smoking, alcohol consumption, and physical activity increased with higher IC levels (p < 0.001 for all). Medical insurance coverage and housing ownership showed modest but statistically significant differences across IC quartiles, while pension insurance coverage and living with children were comparable between groups.

**Table 1:** Demographic and household characteristics of study participants by intrinsic capacity levelpatient demographics and baseline characteristics.

Characteristic	Total	Quartiles of IC				*p-value*
		Quartile 1	Quartile 2	Quartile 3	Quartile 4	
n	10,751	3060	2462	2851	2378	
Age (years)	58.06 ± 8.94	60.48 ± 9.91	58.58 ± 8.90	57.35 ± 8.21	55.25 ± 7.46	0.000
Gender						<0.001
Female	5,676 (52.79)	1745 (57.03)	1432 (58.16)	1493 (52.37)	1006 (42.30)	
Male	5,075 (47.21)	1315 (42.97)	1030 (41.84)	1358 (47.63)	1372 (57.70)	
Education level						<0.001
Middle school or below	9,496 (88.34)	2823 (92.25)	2232 (90.66)	2589 (90.81)	1852 (77.88)	
High school	846 (7.87)	173 (5.65)	143 (5.81)	195 (6.84)	335 (14.09)	
Above high school	409 (3.80)	64 (2.09)	87 (3.53)	67 (2.35)	191 (8.03)	
Marital status						<0.001
Other (unmarried/divorced/widowed)	1,138 (10.58)	468 (15.29)	295 (11.98)	257 (9.01)	118 (4.96)	
Married	9,613 (89.42)	2592 (84.71)	2167 (88.02)	2594 (90.99)	2260 (95.04)	
Living with children						0.165
No	10,288 (95.69)	2922 (95.49)	2347 (95.33)	2724 (95.55)	2295 (96.51)	
Yes	463 (4.31)	138 (4.51)	115 (4.67)	127 (4.45)	83 (3.49)	
Health insurance						0.007
No	636 (5.93)	216 (7.06)	146 (5.93)	156 (5.47)	118 (4.96)	
Yes	10,115 (94.07)	2844 (92.94)	2316 (94.07)	2695 (94.53)	2260 (95.04)	
Pension insurance						0.602
No	9,772 (90.89)	2767 (90.42)	2238 (90.90)	2607 (91.44)	2160 (90.83)	
Yes	979 (9.11)	293 (9.58)	224 (9.10)	244 (8.56)	218 (9.17)	
Smoking						<0.001
No	6,574 (61.15)	1912 (62.48)	1586 (64.42)	1755 (61.56)	1321 (55.55)	
Yes	4,177 (38.85)	1148 (37.52)	876 (35.58)	1096 (38.44)	1057 (44.45)	
Drinking						<0.001
No	7,096 (66.00)	2109 (68.92)	1690 (68.64)	1864 (65.38)	1433 (60.26)	
Yes	3,655 (34.00)	951 (31.08)	772 (31.36)	987 (34.62)	945 (39.74)	
Low physical activity						<0.001
No	7,285 (67.76)	2261 (73.89)	1637 (66.49)	1836 (64.40)	1551 (65.22)	
Yes	3,466 (32.24)	799 (26.11)	825 (33.51)	1015 (35.60)	827 (34.78)	
Moderate physical activity						<0.001
No	8,152 (75.82)	2497 (81.60)	1856 (75.39)	2051 (71.94)	1748 (73.51)	
Yes	2,599 (24.18)	563 (18.40)	606 (24.61)	800 (28.06)	630 (26.49)	
High physical activity						<0.001
No	9,102 (84.66)	2715 (88.73)	2068 (84.00)	2313 (81.13)	2006 (84.36)	
Yes	1,649 (15.34)	345 (11.27)	394 (16.00)	538 (18.87)	372 (15.64)	
Housing ownership						0.014
Non-owner	1,173 (10.91)	373 (12.19)	267 (10.84)	309 (10.84)	224 (9.42)	
Owner	9,578 (89.09)	2687 (87.81)	2195 (89.16)	2542 (89.16)	2154 (90.58)	
Number of children	1.76 ± 1.59	2.08 ± 1.75	1.85 ± 1.61	1.70 ± 1.53	1.35 ± 1.36	<0.001
Household income (10,000 CNY)	8.96 ± 2.51	8.55 ± 2.65	8.84 ± 2.57	8.96 ± 2.47	9.51 ± 2.19	<0.001
Household consumption (10,000 CNY)	9.64 ± 1.13	9.43 ± 1.39	9.61 ± 1.13	9.63 ± 1.00	9.87 ± 0.88	<0.001
Household size (persons)	3.67 ± 1.82	3.65 ± 1.85	3.64 ± 1.84	3.69 ± 1.83	3.71 ± 1.72	0.217

Data were presented as mean±SD or as n (%)

### 3.2. Association between intrinsic capacity and stroke risk

**Fig 2:**
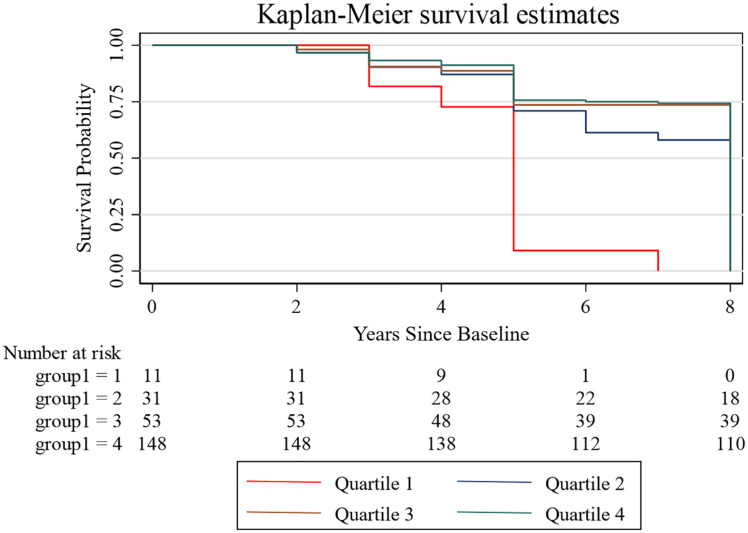
Kaplan–Meier curves for cumulative stroke incidence by IC level. The x-axis represents years since baseline, and the y-axis indicates cumulative stroke-free survival probability.

During a median follow-up period of 7 years, a total of 243 participants (2.26%) experienced incident stroke. The cumulative incidence of stroke declined progressively across increasing quartiles of IC, ranging from 4.84% in Quartile 1 (lowest IC) to 2.15%, 1.09%, and 0.46% in Quartiles 2, 3, and 4, respectively. Kaplan–Meier survival analysis (Fig 2) demonstrated a clear and statistically significant separation of stroke-free survival curves across IC quartiles (*log-rank test, P < 0.001*), with survival probabilities improving stepwise from Quartile 1 to Quartile 4. This graded pattern was evident early in follow-up and became more pronounced over time, indicating that higher baseline IC was associated with a lower cumulative risk of stroke.

To further quantify this association, Cox proportional hazards regression models were employed, with IC modeled both as a categorical variable (quartiles) and a continuous score to examine the robustness of findings (Table 2). In the fully adjusted model (Model 3), and using Quartile 1 as the reference group, participants in Quartile 2 (HR = 0.460; 95% CI: 0.335–0.631), Quartile 3 (HR = 0.244; 95% CI: 0.165–0.363), and Quartile 4 (HR = 0.104; 95% CI: 0.055–0.197) exhibited a progressively lower risk of stroke. The test for trend across quartiles was statistically significant (*P for trend < 0.001*), further supporting a graded, inverse association between IC level and stroke incidence. Consistent with these results, when IC was treated as a continuous variable, each one-point increase in baseline IC score was associated with a 35.1% reduction in stroke risk (HR = 0.649; 95% CI: 0.599–0.702; *P < 0.001*), indicating a robust linear relationship.

**Table 2:** Association between intrinsic capacity and stroke incidence.

Categories	Event, n(%)	Model 1		Model 2		Model 3	
		HR (95%CI)	*P* value	HR (95%CI)	*P* value	HR (95%CI)	*P* value
Total participants	243 (2.26)	0.632 (0.587-0.680)	0.000	0.648 (0.599-0.701)	0.000	0.649 (0.599-0.702)	0.000
Quartile 1	148 (4.84)	Ref.	–	Ref.	–	Ref.	–
Quartile 2	53 (2.15)	0.441 (0.322-0.604)	0.000	0.459 (0.334-0.631)	0.000	0.460 (0.335-0.631)	0.000
Quartile 3	31 (1.09)	0.222 (0.151-0.327)	0.000	0.245 (0.165-0.363)	0.000	0.244 (0.165-0.363)	0.000
Quartile 4	11 (0.46)	0.094 (0.051-0.174)	0.000	0.106 (0.056-0.199)	0.000	0.104 (0.055-0.197)	0.000

Model1: unadjusted for any covariates; Model2: adjusted forsociodemographic factors (age, sex, education, marital status, living arrangement, health insurance, pension status, smoking, alcohol consumption, physical activity, and self-rated health); Model3: Model 2 plus household socioeconomic variables (income, expenditure, household size, housing ownership, number of children); HR:Hazard Ratio, CI: Confidence Interval.

**Fig 3:**
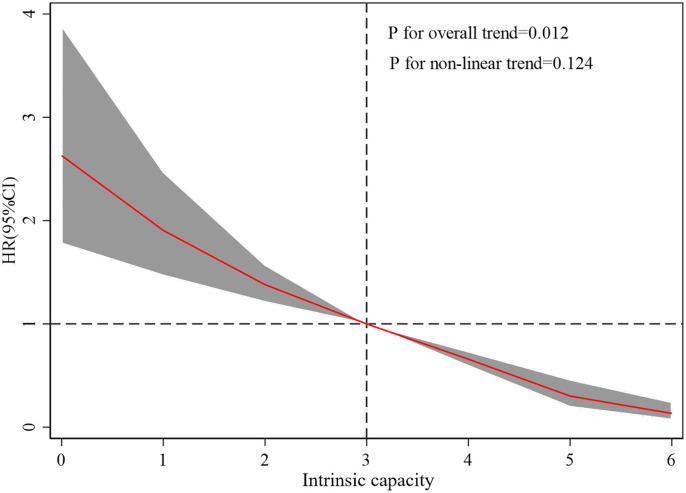
Association of IC and the risk of stroke using a multivariable-adjusted restricted cubic spines model. The thick line indicates adjusted hazard ratio; shaded area represents the 95% confidence interval. The vertical dashed line marks IC = 3, and the horizontal dashed line denotes HR = 1.0.

Restricted cubic spline (RCS) analysis (Fig 3) confirmed an inverse association between IC and stroke risk, with a statistically significant overall trend (*P = 0.012*). Although the test for non-linearity was not statistically significant (*P = 0.124*), the shape of the spline curve suggested a steeper decline in stroke risk at lower IC scores (particularly <3), followed by a plateau at higher levels. These findings indicate that while the relationship is primarily linear, the protective effect of higher IC may be more pronounced among individuals with initially low IC levels.

### 3.3. Subgroup analyses

**Fig 4:**
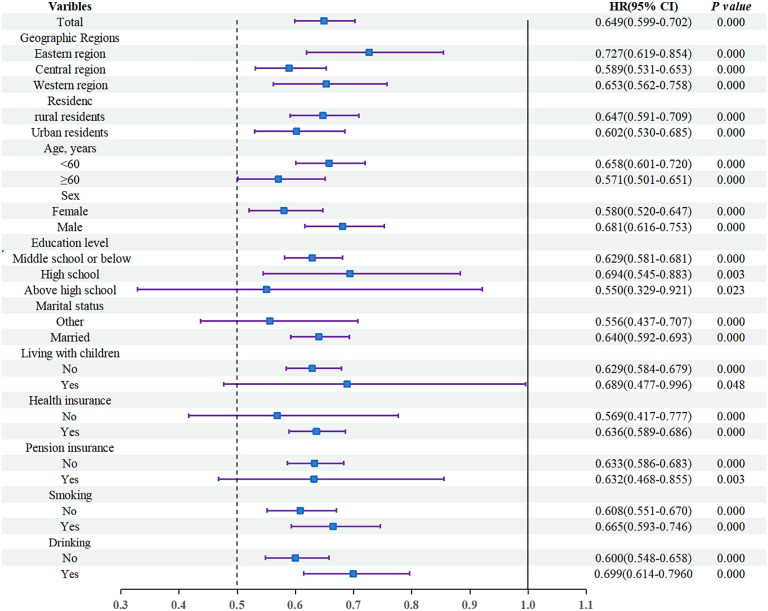
Subgroup and interaction analyses of the association between IC and stroke risk. Hazard ratios (HRs) and 95% confidence intervals (CIs) for stroke associated with higher intrinsic capacity (IC) levels were estimated using multivariable-adjusted Cox proportional hazards models across various subgroups. Models were adjusted for demographic, socioeconomic, behavioral, and health-related covariates. Interaction P-values indicate whether the association between IC and stroke risk significantly differs among subgroups.

To examine the robustness and potential heterogeneity of the relationship between IC and incident stroke, we conducted subgroup analyses stratified by geographic region, urban/rural residence, age, sex, education, marital status, living with children, health behaviors, and insurance status. The findings are visually summarized in the forest plot (Fig 4). Across all subgroups, a higher IC was consistently associated with a significantly reduced risk of stroke, with no subgroup showing a reversal in directionality.

Geographic Regions: The protective effect of IC was most pronounced in the central region (HR = 0.589; 95% CI: 0.531–0.653), followed by the western (HR = 0.653; 95% CI: 0.562–0.758) and eastern regions (HR = 0.727; 95% CI: 0.619–0.854). Residence: Urban residents experienced a stronger stroke risk reduction (HR = 0.602) compared to rural residents (HR = 0.647).

Age and Sex: IC conferred a more substantial protective effect in women (HR = 0.580; 95% CI: 0.520–0.647) and individuals aged 60 years or older (HR = 0.571; 95% CI: 0.501–0.651), suggesting heightened sensitivity and potential for preventive intervention in these groups.

Socio-Demographic Factors: Subgroup analyses by education and marital status revealed significant heterogeneity (*P* < 0.001). Participants with higher education (high school or above) showed stronger protection (HR = 0.550), possibly reflecting better health literacy and self-management capabilities. Those categorized as “other” in marital status (never married, divorced, or widowed) also displayed a pronounced IC-related benefit (HR = 0.556), indicating that individuals with reduced social support may be more vulnerable to IC decline.

Health Behaviors and Insurance: Protective associations were observed in both non-smokers (HR = 0.608) and non-drinkers (HR = 0.600), suggesting that healthy lifestyle habits may synergistically enhance the effect of IC on stroke prevention. Similar trends were noted across strata defined by co-residence with children, and insurance participation (both medical and pension).

### 3.4. Sensitivity analyses

To evaluate the robustness of the association between IC and stroke risk, we performed two sensitivity analyses. First, we excluded participants with cognitive impairment at baseline (n = 968) to minimize potential confounding from pre-existing neurological decline (Table 3). The association between IC and incident stroke remained consistent and statistically significant across all models. In the fully adjusted model (Model 3), compared with participants in the lowest IC quartile (Q1), those in Quartile 2 had a 56.6% lower risk of stroke (HR = 0.434; 95% CI: 0.311–0.606; *P < 0.001*), those in Quartile 3 had a 77.4% lower risk (HR = 0.226; 95% CI: 0.150–0.341; *P < 0.001*), and those in Quartile 4 had a risk reduction of approximately 90.8% (HR = 0.092; 95% CI: 0.048–0.177; *P < 0.001*).

**Table 3:** Association between intrinsic capacity and stroke after excluding cognitively impaired participants.

Categories	Event, n(%)	Model 1		Model 2		Model 3	
		HR (95%CI)	*P* value	HR (95%CI)	*P* value	HR (95%CI)	*P* value
Total participants	209 (2.14)	0.547 (0.499-0.599)	0.000	0.546 (0.493-0.605)	0.000	0.543 (0.490-0.601)	0.000
Quartile 1	114 (5.14)	Ref.	–	Ref.	–	Ref.	–
Quartile 2	53 (2.22)	0.428 (0.309-0.593)	0.000	0.435 (0.312-0.607)	0.000	0.434 (0.311-0.606)	0.000
Quartile 3	31 (1.11)	0.213 (0.143-0.317)	0.000	0.227 (0.150-0.343)	0.000	0.226 (0.150-0.341)	0.000
Quartile 4	11 (0.46)	0.088 (0.047-0.164)	0.000	0.094 (0.049-0.179)	0.000	0.092 (0.048-0.177)	0.000

In a second sensitivity analysis, we excluded individuals aged ≥80 years at baseline (n = 202) to assess whether extreme age influenced the observed associations (Table 4). The findings remained robust and directionally consistent. In Model 3, the hazard ratios for stroke incidence were 0.466 (95% CI: 0.338–0.642) for Quartile 2, 0.250 (95% CI: 0.168–0.372) for Quartile 3, and 0.106 (95% CI: 0.056–0.200) for Quartile 4, relative to Quartile 1.

**Table 4:** Association between intrinsic capacity and stroke after excluding participants aged ≥80 years.

Categories	Event, n(%)	Model 1		Model 2		Model 3	
		HR (95%CI)	P value	HR (95%CI)	P value	HR (95%CI)	P value
Total participants	235 (2.23)	0.629 (0.584-0.679)	0.000	0.649 (0.599-0.703)	0.000	0.650 (0.600-0.704)	0.000
Quartile 1	141 (4.82)	Ref.	–	Ref.	–	Ref.	–
Quartile 2	52 (2.15)	0.444 (0.323-0.610)	0.000	0.464 (0.337-0.640)	0.000	0.466 (0.338-0.642)	0.000
Quartile 3	31 (1.09)	0.224 (0.152-0.331)	0.000	0.250 (0.168-0.372)	0.000	0.250 (0.168-0.372)	0.000
Quartile 4	11 (0.46)	0.095 (0.051-0.175)	0.000	0.107 (0.057-0.202)	0.000	0.106 (0.056-0.200)	0.000

## 4. Discussion

In this large, nationally representative prospective cohort study of 10,751 middle-aged and older Chinese adults, we found that higher IC scores were consistently associated with a reduced risk of incident stroke. This inverse association remained robust and statistically significant after adjustment for sociodemographic, behavioral, and health-related covariates. Kaplan–Meier survival analysis, Cox proportional hazards models, and restricted cubic spline (RCS) analysis collectively demonstrated a graded, predominantly linear inverse relationship between IC and stroke risk, with the steepest decline observed at lower IC levels. The findings were further supported by consistent results across multiple sensitivity and subgroup analyses. These results provide empirical support for the WHO's healthy aging framework, which conceptualizes IC as a dynamic measure of an individual's functional reserve that can help anticipate future health events before clinical disease manifests. Rather than confirming predictive utility, our findings support IC as a potential functional indicator or risk stratification tool for stroke.

Stroke incidence in China continues to rise rapidly—annual increases of approximately 8.8% for first strokes among adults aged ≥40 have been reported [41]. Conventional stroke risk assessments often rely on blood pressure [42], glycemic indices [43], triglyceride-glucose index [44], BMI [45], visceral adiposity and atherosclerosis markers [46,47], which typically detect disease only after substantial pathology develops. In contrast, IC offers a multidimensional, function-based assessment that may capture early subclinical decline across physical, cognitive, and psychosocial domains [48]. As such, IC may reflect a preclinical stage of vulnerability that precedes the onset of overt cerebrovascular damage—making it a conceptually grounded tool for upstream prevention. Our results highlight the potential value of IC as a complementary metric for early risk identification, and support ongoing efforts to shift from disease-centered to function-centered models of chronic disease prevention and management [49].

Previous studies using UK Biobank data have demonstrated that IC is associated with cardiovascular and all-cause mortality [50], while research using CHARLS has linked IC decline to multimorbidity and disability [51]. Previous studies using UK Biobank data have demonstrated that IC is associated with cardiovascular and all-cause mortality [52], while research using CHARLS has linked IC decline to multimorbidity and disability [53]. A recent study by Guo [54] also examined intrinsic capacity trajectories and incident cardiovascular disease (CVD) using the CHARLS dataset. While their study identified unfavorable IC trajectories as predictors of increased CVD risk in Chinese adults aged ≥60 years, our study differs in several key aspects. First, we focused specifically on stroke as a distinct and clinically important outcome, rather than general CVD. Second, we included a broader age range (≥45 years), enabling the identification of functional risk signals in middle-aged individuals before entry into advanced aging. Third, while Guo et al. employed trajectory modeling across multiple waves, our approach—based on baseline IC quartiles—offers a simpler and potentially more pragmatic framework for early screening in primary care. Together, these studies complement one another by providing convergent evidence on the role of IC in vascular risk across different stages of aging and methodological approaches. Our findings extend this literature by showing that stroke, as a highly disabling and fatal condition, is closely associated with multidomain declines in function even before clinical diagnosis.

Subgroup analyses revealed that the protective association between higher IC and stroke incidence was particularly evident among women, older adults, urban residents, individuals living in central China, and those without a history of smoking or alcohol consumption. These variations may reflect underlying heterogeneity in physiological reserves, cumulative life-course exposures, and behavioral or environmental risk factors [55–57]. For example, hormonal and metabolic shifts in older adults, particularly women, may heighten susceptibility to neurovascular injury [58]. While urban residents may have better access to care, lifestyle-related stress and reduced physical activity may attenuate the benefits of IC [59,60]. Central regions of China, which are undergoing rapid healthcare transitions, may also represent areas where declines in IC more directly translate into adverse health outcomes, including stroke [61]. Individuals with smoking or alcohol use may experience additive physiological damage that diminishes the protective effects of IC, whereas healthier lifestyle profiles may enable individuals to realize the full benefit of preserved function [62,63]. Additionally, higher education and stable marital status likely enhance cognitive reserve and social support, which may reinforce the advantages of higher IC [64,65].

Compared to traditional metabolic biomarkers, which capture limited physiological domains, IC represents a holistic measure that integrates mobility, cognition, vitality, sensory capacity, and psychological resilience. Our findings support the feasibility of incorporating IC into population-level screening or primary care risk assessments, especially for aging adults who may not yet meet criteria for overt disease diagnosis. The consistency of associations across sensitivity analyses—such as excluding those with baseline cognitive impairment and individuals aged ≥80 years—adds further credibility to IC’s role as a stable, functional indicator of stroke vulnerability.

These results align with emerging frameworks in aging research that advocate for the use of function-based measures in disease prevention and health promotion. As highlighted by The Lancet Healthy Longevity, IC assessments can inform early-stage, personalized interventions to promote healthy aging and prevent loss of independence [66]. Our findings contribute to this paradigm by showing that IC may help identify individuals at increased stroke risk prior to clinical onset—especially in settings where resources for advanced diagnostic testing are limited. This supports the conceptual framing of IC as a modifiable functional reserve that, if preserved or improved, could meaningfully reduce the burden of stroke and promote longevity with independence. Importantly, we do not claim IC to be a definitive predictor, but rather a valuable marker of early physiological and functional decline that merits attention in public health and clinical practice. Thus, early intervention to maintain or enhance IC may serve as a promising strategy to mitigate stroke burden in aging populations.

## 5. Limitations

While this study provides valuable insights into the association between intrinsic capacity (IC) and stroke risk in a large, nationally representative cohort of Chinese middle-aged and older adults, several limitations should be considered. First, stroke events were identified through self-reported physician diagnoses, which may be subject to recall bias or misclassification, potentially leading to under- or overestimation of true incidence rates. Second, some IC components—particularly vitality and psychological well-being—were measured using subjective self-reported items rather than objective clinical or performance-based assessments, which may reduce measurement precision and introduce reporting bias. Third, due to the observational nature of the study, causal relationships cannot be established, and residual confounding or reverse causality cannot be fully excluded, including potential confounding from baseline cardiovascular comorbidities such as heart disease, despite adjustment for multiple health-related covariates. Fourth, the CHARLS cohort reflects the specific sociocultural and healthcare context of China, which may limit the generalizability of our findings to other populations with different health systems, cultural norms, or risk factor profiles. Finally, we did not perform formal predictive modeling or external validation; thus, although IC was associated with stroke risk, we cannot infer its predictive accuracy or clinical utility in individual risk stratification.

Future studies should incorporate clinically validated stroke diagnoses, use objective functional assessments, and apply formal risk prediction models to evaluate the prognostic value of IC. Longitudinal and interventional research is also warranted to determine whether preserving or enhancing IC can causally reduce stroke risk and promote healthy aging.

## 6. Conclusion

In this large, nationally representative cohort of Chinese middle-aged and older adults, higher levels of IC were significantly associated with a lower risk of incident stroke. The inverse association remained robust after adjustment for sociodemographic and health-related factors and was particularly pronounced among women, older individuals, urban residents, those living in central China, and individuals without smoking or alcohol use behaviors.

These findings extend previous research by highlighting the potential utility of IC as a multidimensional, function-based indicator for early stroke risk identification. Given that IC captures physical, cognitive, and psychosocial domains, it may provide a more comprehensive assessment of health vulnerability than conventional risk factors.

By identifying individuals at elevated stroke risk before overt disease onset, IC may inform timely preventive strategies in aging populations. Future studies should further evaluate its clinical utility across diverse settings and assess whether interventions that maintain or enhance IC can effectively reduce stroke burden.

## Abbreviations

IC: Intrinsic Capacity
CHARLS: China Health and Retirement Longitudinal Study
WHO: World Health Organization
HR: Hazard Ratio
CI: Confidence Interval
RCS: Restricted Cubic Spline
BMI: Body Mass Index
CES-D: Center for Epidemiologic Studies Depression Scale
DALYs: Disability-Adjusted Life Years
GBD: Global Burden of Disease
UK: Biobank United Kingdom Biobank (Prospective Cohort Study)
VAI: Visceral Adiposity Index
CVAI: Chinese Visceral Adiposity Index
ANOVA: Analysis of Variance
